# Do dysfunctional coping modes mediate the relationship between perceived parenting style and disordered eating behaviours?

**DOI:** 10.1186/s40337-016-0123-1

**Published:** 2016-11-02

**Authors:** Jessica M. Brown, Stephanie Selth, Alexander Stretton, Susan Simpson

**Affiliations:** 1 School of Psychology, University of Adelaide, North Tce, Adelaide, 5005 SA Australia; 2 Psychology Clinic, School of Psychology, Social Work, and Social Policy, University of South Australia, Magill Campus, GPO Box 2471, Adelaide, 5001 SA Australia; 3 School of Education, Arts and Social Sciences, University of South Australia, Adelaide, SA Australia

**Keywords:** Eating disorders, Schema modes, Coping, Parenting, Schema Therapy

## Abstract

**Background:**

Preliminary studies suggest that both childhood experiences and coping behaviours may be linked to eating disorder symptoms.

**Methods:**

In this study maladaptive schema coping modes were investigated as mediators in the relationship between perceived negative parenting and disordered eating. A total of 174 adults with eating and/or body image concerns completed questionnaires measuring parenting experiences, schema modes, and disordered eating behaviours.

**Results:**

Perfectionistic Overcontroller, Self-Aggrandiser, Compliant Surrenderer, Detached Protector and Detached Self-Soother coping modes partially explained the variance in the relationships between perceived negative parenting experiences and the behaviours of restricting and compensation (purging and overexercising).

**Conclusions:**

Our findings suggest that Overcompensatory, Avoidant and Surrender coping mechanisms all appear to play a role in the maintenance of eating disorder symptoms, and that there are multiple complex relationships between these and Early Maladaptive Schemas that warrant further investigation.

## Plain english summary

In this study, we investigated whether perceived childhood parenting experiences and coping mechanisms played a role in the development of eating disorder symptoms in a group of 174 participants who indicated that they had difficulties with eating and/or body image. The maladaptive coping mechanisms of perfectionism, avoidance, and compliance partially explained the relationship between perceived negative parenting experiences and restriction/purging/overexercising. Schema Therapy may therefore be an appropriate treatment that can address developmental factors associated with parenting, alongside here-and-now maintaining factors, including core beliefs and coping style.

## Background

Eating disorders are considered one of the most difficult psychopathologies to treat, due to high levels of complexity, ego-syntonicity, chronicity, and heterogeneity [1, 48]. Comorbidity with other mental health disorders is high, including anxiety, mood and substance misuse disorders [5, 11, 34] and personality disorders [8]. Although Cognitive Behavioural Therapy (CBT) is the treatment of choice for eating disorders (EDs), a significant number of sufferers do not respond [2]. Anorexia Nervosa in particular remains exceptionally difficult to treat, with Enhanced CBT outcome trials reporting low remission and high attrition rates [9, 10, 18, 19]. Given the limited efficacy of maintenance models such as CBT in the treatment of EDs, especially for those with higher comorbidity and severity, deeper level factors are now a focus of the ED literature [22], and there is a need for new and innovative therapeutic models to improve our conceptualization and treatment of the eating disorders [13, 55].

Schema Therapy encapsulates developmental, maintenance, and deeper level themes and was developed to conceptualise complex psychopathology with high comorbidity, including personality disorders [57]. Early maladaptive schemas (EMS) are defined as unconditional, implicit, and irrefutable cognitive beliefs that result from unmet core needs and repeated negative experiences with significant others during childhood and adolescence [57]. EMS act as frameworks for understanding interpersonal relationships and life experiences [28]. Schema modes are defined as the states or parts of the personality and coping mechanisms that manifest moment-to-moment. Further, there is preliminary, though limited, empirical support for the application of the schema model to ED populations [29, 40, 41]. Specifically, research has linked EMS and schema processes to ED pathology, and ED populations have been found to score higher on EMS [22, 25, 52] and schema modes [45, 49] than non-clinical populations. Preliminary studies suggest that the Compliant Surrenderer, and two avoidant coping modes, Detached Self-Soother and Detached Protector, appear to be higher in the ED population than in non-clinical populations [45] and other clinical groups [49].

Preliminary studies have also reported a link between negative parenting experiences – particularly emotional abuse and invalidation – and ED pathology [17, 24, 25, 47, 53]. Further, emerging evidence suggests EMS and schema coping processes may explain the link between adverse childhood experiences and the onset of ED pathology [15, 36, 47]. Turner et al. [47] found high maternal over-protection and low paternal care were significantly related to the ‘Defectiveness’ and ‘Dependence’ schemas. In turn, Defectiveness and Dependence mediated the relationship between parental bonding and ED symptoms. Similarly, Deas et al. [15] found participants with AN had significantly more EMS – particularly perfectionistic schemas - and perceived their parents as less caring and more controlling than healthy controls. Sheffield et al. [36] also found the variables of social control (an over-compensation process) and behavioural-somatic avoidance (an avoidance process) to partially mediate correlations between particular negative parenting experiences and ED pathology. This is the first study to our knowledge that has explored the role of dysfunctional coping modes in explaining the relationship between parenting and ED behaviours.

The current study aimed to extend the literature by investigating whether dysfunctional coping modes mediate the relationship between perceived negative parenting and the ED behaviours of restricting, binging, and overcompensation (purging and overexercising). Schema coping modes were selected as a key variable since they amalgamate EMS and schema processes into a unified construct, both of which have been empirically linked to ED pathology [22, 25, 27]. The three overarching coping mechanisms that encompass the coping modes are ‘Surrender’ (Compliant Surrenderer mode), ‘Overcompensation’ (Perfectionistic Overcontroller mode, Self Aggrandiser Mode, Bully and Attack Mode), and ‘Avoidance’ (Detached Protector, Detached Self-Soother). Based on the aforementioned preliminary research, it is hypothesized that perceived negative parenting experiences will be positively correlated with both ED behaviours and dysfunctional coping modes, with behaviours and coping modes also positively correlated. It is further hypothesized that dysfunctional coping modes will mediate the relationship between (a) perceived negative parenting and (b) ED behaviours.

## Method

### Participants

Given the high prevalence of ED behaviours (full and subthreshold) within the general population [20] the current study recruited participants through advertisements on ED and psychology pages and groups on LinkedIn and Facebook, requesting individuals aged 18 to 65 who held concerns about their eating behaviours, eating attitudes, and/or body image to take part in an online survey exploring how perceptions of parenting may impact the development of specific coping modes which may in turn influence eating and body image. The advertisement included a link to the survey which participants were invited to complete online through Survey Monkey. In all, 187 participants completed the study questionnaire. Thirteen participants were excluded as they failed to meet the key selection criteria of eating and/or body image concerns, as measured by the Eating disorder diagnostic scale (EDDS; [44]).

The final sample included 174 participants ranging in age from 18 to 65 years (m = 28.6, SD = 9.2). Participant demographics, including diagnoses of EDs, are presented in Table 1. Diagnoses were made using self-report responses on the EDDS following the Diagnostic and Statistical Manual of Mental Disorders (5^th^ ed.; DSM-5; [3]) criteria. However, eating disorder behaviours rather than diagnoses were used as outcome variables. 129 participants met the criteria for an Other Specified Feeding or Eating Disorder (OSFED), with 14.4 % (25) of these reporting a BMI of 18.5 or less. Along with the fact that none of the sample met criteria for full threshold Bulimia or Binge Eating Disorder, it appears that a high proportion of the sample were engaging in significant food restriction, with binge eating being underrepresented.

**Table 1:** Participant Demographic Information (*N* = 174)

	Frequency	Percentage
Nationality		
Australian	76	43.7
American	20	11.5
New Zealander	12	6.9
Canadian	11	6.3
Hispanic	5	2.9
Asian	10	5.7
British	34	19.5
Mainland European	6	3.4
Sex		
Male	12	7
Female	162	93
Currently Consulting a Mental Health Professional		
Yes	53	30.5
No	121	69.5
Consulted a Mental Health Practitioner in the Past		
Yes	118	67.8
No	56	32.2
Eating Disorder Diagnostic Scale Diagnosis		
No diagnosis	32	18.4
Full threshold AN	9	5.2
OSFED	133	76.4

*AN* Anorexia Nervosa, *OFSED* Other Specified Feeding or Eating Disorder

*Note: Eating disorder diagnoses were determined using criteria outlined by the Diagnostic and Statistical Manual of Mental Disorders, 5^th^ Edition [3] using participant responses to the Eating Disorder Diagnostic Scale [44]

### Instruments

#### Young Parenting Inventory-Revised (YPI-R; [37])

The YPI-R assesses perceived negative parenting. The YPI-R is a 37-item questionnaire measuring perceived parenting styles, namely: emotionally depriving; overprotective; belittling; perfectionist; pessimistic/fearful; controlling; emotionally inhibited; punitive; and conditional/narcissistic. Items relating to each style were rated using two 6-point Likert scales – one for perceptions of the mother and one for the father. Scores for each style were determined by summing the answers of items associated with each style, with higher scores indicating greater perceptions of that parenting style occurring. The YPI-R has been shown to be a reliable and valid measure of perceived parenting [37].

#### Schema Mode Inventory (SMI, [56])

The SMI is a 124-item self- report measure assessing: child modes (vulnerable and emotional states), dysfunctional coping modes (Compliant Surrenderer, Detached Self-Soother, Detached Protector, Self-aggrandizer, and Bully/attack), parent modes (the Punitive and Demanding modes), and adaptive modes (the Healthy Adult). In this study only the dysfunctional coping modes were explored. In addition, eleven items were added to the SMI to measure the Perfectionistic Overcontroller coping mode. These items had been removed from the original SMI after analyses showed it did not emerge as a separate factor. However, as original analyses were not conducted specifically with an ED population, it was considered prudent to re-include it in this study due to the clinical observation that perfectionism is typical in this population [15, 39, 50, 51]. Participants rated how frequently each statement applied to them on a 6-point Likert scale, ranging from “never or almost never” to “all of the time”. Scores for each dysfunctional coping mode were calculated using the mean score of all items related to that mode. Lobbestael et al. [26]) indicated good internal consistencies (Cronbach’s α ranging from .76 to .96), and test-retest validity (mean ICC =. 84).

#### Eating Disorder Diagnostic Scale (EDDS; [44])

The EDDS is a 22-item self-report measure of full and sub-threshold Anorexia Nervosa, Bulimia Nervosa, and Binge Eating Disorders. Research evidence shows the EDDS has high internal consistency (α = .89) and test-retest reliability (0.87) and is a valid measure when used with both adolescents and adults [43, 44]. In addition, subscales on the Eating Disorder Examination [12] correlate moderately with total EDDS scores, and concordance between diagnoses made by these methods is high, ranging from 93 % for Binge Eating Disorder to 99 % for Anorexia Nervosa [44]. Participants were asked to estimate over the past 3 months how often each week on average they had engaged in binge-eating, excessive exercise, vomiting, fasting and used laxatives or diuretics (range: 0–14). As the current study used a general population sample, ED behaviours (restriction, binge-eating, compensatory behaviours (over-exercise, purging), rather than diagnoses, were used as outcome variables.

### Procedure

#### Design

The study used a quantitative, cross-sectional survey design.

### Statistical power

As the Schema Mode Inventory (SMI) has not been validated for use with ED populations, statistical power was estimated from previous studies using the SMI with personality disorder samples that reported moderate effects (d = 0.56: [33]). Given a medium effect size, power of .80, and an alpha level of .05, G*Power [16] software calculated a minimum of 129 participants were required for the study.

### Ethical considerations

The University of South Australia’s Human Research Ethics Committee approved the study.

### Statistical analyses

Descriptive statistics and correlational analyses were conducted using the Statistical Packages for the Social Sciences (SPSS) version 21. Bootstrap mediation methods outlined by Preacher and Hayes [31] were used to analyse the mediating role of dysfunctional coping modes in the relationship between perceived negative parenting experiences and disordered eating. This involved calculating the direct effect (c) of perceived negative parenting experiences (a) on disordered eating (b), followed by calculating the total effect (c’) of that same relationship, modelling in the mediator variable, and finally calculating the indirect effect (a x b) of the relationship through the mediator variable (dysfunctional coping mode). The mediation classification decision tree developed by Zhao et al. [58] was used to determine the type of mediation and the theoretical implications. Five thousand bootstrap re-samples using the bias-corrected and accelerated bootstrap method were applied to all indirect effect analyses, which were conducted using the AMOS Graphics Software [4], Version 20. This method affords a number of benefits when compared to the Sobel test, including increased statistical power, the ability to detect indirect effects when direct effects are not detected, and the ability to conduct significance tests on the indirect effect without the need for normally distributed variables [58]. Complementary mediation is synonymous with partial mediation [58].

## Results

### Descriptive statistics

Descriptive statistics including the mean and standard deviations for each variable and internal consistency analysis are included in Table 2.

**Table 2:** Descriptive statistics for the dysfunctional coping modes (SMI, [56]), perceptions of negative parenting experiences (YPI-R; [37]) and eating disorder behaviours (EDDS; [44])

	α	Mean (*N* = 174)	SD
Perfectionistic Overcontroller	.80	3.73	.83
Self-Aggrandiser	.77	2.80	.75
Bully and Attack	.71	1.98	.62
Compliant Surrenderer	.84	3.72	.97
Detached Protector	.90	2.99	1.05
Detached Self-Soother	.75	3.68	1.06
Overprotective father	.80	2.46	1.06
Pessimistic/fearful father	.79	2.86	1.30
Controlling father	.79	2.23	1.27
Conditional/narcissistic father	.83	3.14	1.40
Emotionally depriving father	.93	3.55	1.48
Belittling mother	.94	2.22	1.31
Emotionally inhibited mother	.84	3.14	1.50
Punitive mother	.83	3.32	1.52
Conditional/narcissistic mother	.82	3.27	1.38
Emotionally depriving mother	.92	2.92	1.31
Perfectionistic mother	.64	3.75	1.20
Restricting	-	3.22	4.02
Binging	-	2.39	2.90
Compensatory Behaviour	-	4.93	6.78

### Correlational analyses

Pearson correlational analyses between perceived negative parenting experiences, specific coping modes, and disordered eating are presented in Table 3. Both restricting and compensatory behaviour were weakly correlated with a variety of different perceived negative parenting experiences, however binge eating was not correlated at all and was thus excluded from the mediation analysis. All dysfunctional coping styles, with the exception of Bully/attack, were weakly to moderately correlated with several different parenting experiences, with the strongest correlations seen between the Perfectionistic Overcontroller and both controlling and conditional/narcissistic father, Detached Self-soother with belittling, emotionally depriving and emotionally inhibited mother, and both the Compliant Surrenderer and Detached Protector with emotionally inhibited mother. Restriction was weakly to moderately correlated with all dysfunctional coping modes except Bully/attack, with the strongest correlation being with Perfectionistic Overcontroller. Binge eating was only weakly correlated with a single mode – the Detached Protector. Finally, compensatory behaviour was strongly correlated with the Detached Protector, with weak to moderate correlations with all other dysfunctional coping modes.

**Table 3:** Correlations between perceived negative parenting experiences and mediator and outcome variables

Perceived negative parenting experience	Correlations between mediation variables								
	Restricting	Compensatory behaviour	Binging	Perfectionistic overcontroller	Self aggrandiser	Compliant surrenderer	Detached self-soother	Detached protector	Bully and attack
Overprotective father	.281^a^	.090	-.026	.196^a^	.182^b^	.162^b^	.293^a^	.156^b^	.140
Pessimistic/fearful father	.209^a^	.042	.041	.212^a^	.151^b^	.140	.268^a^	.192^b^	.129
Controlling father	.201^a^	.113	-.046	.305^a^	.147	.248^a^	.260^a^	.279^a^	.183^b^
Conditional/narcissistic father	.282^a^	.205^a^	.005	.323^a^	.276^a^	.196^a^	.271^a^	.203^a^	.181^b^
Emotionally depriving father	.109	.113	.072	.220^a^	.082	.278^a^	.231^a^	.261^a^	.120
Belittling mother	.197^a^	.242^a^	-.037	.258^a^	.154^b^	.280^a^	.336^a^	.282^a^	.185^b^
Emotionally inhibited mother	.250^a^	.265^a^	-.020	.261^a^	.126	.305^a^	.307^a^	.299^a^	.102
Punitive mother	.192^b^	.216^a^	-.155	.256^a^	.198^a^	.273^a^	.238^a^	.184^b^	.173^b^
Conditional/narcissistic mother	.280^a^	.280^a^	-.092	.207^a^	.220^a^	.258^a^	.284^a^	.172^b^	.183^b^
Emotionally depriving mother	.177^b^	.191^b^	-.090	.249^a^	.046	.261^a^	.299^a^	.276^a^	.109
Perfectionistic mother	.181^b^	.226^a^	.041	.138	.330^a^	.257^a^	.207^a^	.173^b^	.172^b^
Restricting	-	.508^a^	-.054	.431^a^	.195^a^	.347^a^	.286^a^	.376^a^	.026
Compensatory Behaviour	.508^a^	-	.139	.429^a^	.224^a^	.361^a^	.368^a^	.518^a^	.253^a^
Binging	-.054	.139	-	-.104	-.077	-.004	.103	.152^b^	.008

^a^Significant at the .01 level

^b^Significant at the .05 level

### Model and mediation analyses

In the first instance a model was run to assess the percentage of variance explained in disordered eating by the schema modes in the present study. Together the six schema modes explained 18 % of the variance in restricting, 23 % of the variance in binging and 23 % of the variance in purging/overexercising.

All significant correlations between perceived negative parenting variables and ED behaviours were investigated to determine whether dysfunctional coping modes mediated the relationships. Given the large number of correlations performed a more stringent significance level (*p* < .01) was set to determine inclusion of specific perceived negative parenting styles in the mediation analyses. As Table 4 shows, the relationships between perceived negative parenting experiences and restricting were mediated by both the Perfectionistic Overcontroller and Self-Aggrandiser, with the former fully mediating four relationships between perceived parenting styles and restricting. The Perfectionistic Overcontroller and Self-Aggrandiser also mediated the relationships between perceived negative parenting and compensatory behaviour, although the Self-Aggrandiser explained very little variance. The coping mode of Bully/attack was not correlated with any perceived negative parenting variables and was thus excluded from mediational analysis. The Compliant Surrenderer was found to mediate the relationships between negative parenting and both restricting and compensatory behaviour. The Detached Protector and Detached Self-Soother further mediated the relationships between perceived negative parenting experiences and both restriction and compensatory behaviour.

**Table 4:** Perceived negative parenting experiences and disordered eating mediated by schema modes

	Direct effect (c)	Total effect (c’)	Indirect effect (a x b)	95 % CI	Type of mediation
	Perceived negative parenting and restricting mediated by perfectionistic controller				
Overprotective father	.290^a^	.211^a^	.079^a^	.028 / .148	Complementary
Pessimistic/fearful father	.214^a^	.127	.087^a^	.030 / .157	Full
Controlling father	.204^a^	.077	.128^a^	.070 / .201	Full
Conditional/narcissistic father	.284^a^	.163^b^	.122^a^	.065 / .195	Complementary
Belittling mother	.205^a^	.112	.093^a^	.036 / .166	Full
Emotionally inhibited mother	.251^a^	.144^b^	.107^a^	.049 / .182	Complementary
Punitive mother	.200^a^	.096	.104^a^	.048 / .177	Full
Conditional/narcissistic mother	.285^a^	.202^a^	.083^a^	.029 / .151	Complementary
	Perceived negative parenting and compensatory behavior mediated by perfectionistic controller				
Emotionally depriving mother	.191^b^	.090	.101^a^	.045 / .175	Full
Conditional/narcissistic mother	.280^a^	.199^a^	.080^a^	.026 / .153	Complementary
Punitive mother	.216^a^	.114	.102^a^	.044 / .184	Full
Emotionally inhibited mother	.265^a^	.165^b^	.101^a^	.044 / .179	Complementary
Belittling mother	.242^a^	.141^b^	.101^a^	.042 / .178	Complementary
Perfectionistic mother	.226^a^	.170^b^	.056^b^	.004 / .127	Complementary
	Perceived negative parenting experiences and restricting mediated by Self-Aggrandiser				
Overprotective father	.290^a^	.262^a^	.028^b^	.002 / .083	Complementary
Pessimistic/fearful father	.214^a^	.188^b^	.026^b^	.002 / .082	Complementary
Controlling father	.204^a^	.178^b^	.026^b^	.001 / .085	Complementary
Conditional/narcissistic father	.284^a^	.249^a^	.035	-.003 / .100	No mediation
Belittling mother	.205^a^	.175^b^	.030^b^	.003 / .081	Complementary
Emotionally inhibited mother	.251^a^	.228^a^	.023^b^	.000 / .074	Complementary
Punitive mother	.200^a^	.167^b^	.032^b^	.004 / .086	Complementary
Conditional/narcissistic mother	.285^a^	.253^a^	.032^b^	.001 / .088	Complementary
	Perceived negative parenting experiences and compensatory behaviour mediated by Self-Aggrandiser				
Emotionally depriving mother	.191^b^	.181^b^	.010	-.018 / .055	No mediation
Conditional/narcissistic mother	.280^a^	.242^a^	.038^b^	.006 / .091	Complementary
Punitive mother	.216^a^	.179^b^	.037^b^	.006 / .092	Complementary
Emotionally inhibited mother	.265^a^	.241^a^	.024^b^	.000 / .075	Complementary
Belittling mother	.242^a^	.213^a^	.030^b^	.003 / .078	Complementary
Perfectionistic mother	.226^a^	.170^b^	.056^b^	.008 / .122	Complementary
	Perceived negative parenting experiences and restricting mediated by Compliant Surrenderer				
Overprotective father	.290^a^	.240^a^	.050^b^	.002 / .119	Complementary
Pessimistic/fearful father	.214^a^	.171^b^	.043^b^	.002 / .105	Complementary
Controlling father	.204^a^	.122	.082^a^	.028 / .161	Full
Conditional/narcissistic father	.284^a^	.227^a^	.058^b^	.011 / .132	Complementary
Belittling mother	.205^a^	.131	.074^a^	.022 / .160	Full
Emotionally inhibited mother	.251^a^	.153^b^	.098^a^	.042 / .191	Complementary
Punitive mother	.200^a^	.111	.089^a^	.039 / .166	Full
Conditional/narcissistic mother	.285^a^	.203^a^	.081^a^	.032 / .161	Complementary
	Perceived negative parenting experiences and compensatory behaviour mediated by Compliant Surrenderer				
Emotionally depriving mother	.191^b^	.104	.087^a^	.037 / .166	Full
Conditional/narcissistic mother	.280^a^	.200^a^	.080^a^	.034 / .146	Complementary
Punitive mother	.216^a^	.127	.089^a^	.043 / .156	Full
Emotionally inhibited mother	.265^a^	.171^b^	.094^a^	.047 / .170	Complementary
Belittling mother	.242^a^	.153^b^	.089^a^	.040 / .158	Complementary
Perfectionistic mother	.226^a^	.142	.083^a^	.036 / .152	Full
	Perceived negative parenting experiences and restricting mediated by Detached Protector				
Overprotective father	.290^a^	.234^a^	.056^b^	.009 / .123	Complementary
Pessimistic/fearful father	.214^a^	.146^b^	.068^a^	.022 / .138	Complementary
Controlling father	.204^a^	.103	.101^a^	.046 / .179	Full
Conditional/narcissistic father	.284^a^	.218^a^	.067^a^	.019 / .139	Complementary
Belittling mother	.205^a^	.095	.110^a^	.054 / .186	Full
Emotionally inhibited mother	.251^a^	.144^b^	.107^a^	.049 / .190	Complementary
Punitive mother	.200^a^	.133	.066^a^	.016 / .137	Full
Conditional/narcissistic mother	.285^a^	.220^a^	.064^a^	.016 / .135	Complementary
	Perceived negative parenting experiences and compensatory behaviour mediated by Detached Protector				
Emotionally depriving mother	.191^b^	.052	.139^a^	.072 / .226	Full
Conditional/narcissistic mother	.280^a^	.196^a^	.083^b^	.017 / .157	Complementary
Punitive mother	.216^a^	.125	.091^a^	.023 / .172	Full
Emotionally inhibited mother	.265^a^	.121	.144^a^	.079 / .224	Full
Belittling mother	.242^a^	.104	.138^a^	.074 / .214	Full
Perfectionistic mother	.226^a^	.140^b^	.085^a^	.022 / .157	Complementary
	Perceived negative parenting experiences and restricting mediated by Detached Self-Soother				
Overprotective father	.290^a^	.227^a^	.066^a^	.027 / .132	Complementary
Pessimistic/fearful father	.214^a^	.147	.068^a^	.027 / .130	Full
Controlling father	.204^a^	.136	.069^a^	.028 / .134	Full
Conditional/narcissistic father	.284^a^	.223^a^	.062^a^	.023 / .124	Complementary
Belittling mother	.205^a^	.125	.080^a^	.033 / .144	Full
Emotionally inhibited mother	.251^a^	.178^b^	.074^a^	.029 / .143	Complementary
Punitive mother	.200^a^	.141	.058^a^	.020 / .118	Full
Conditional/narcissistic mother	.285^a^	.221^a^	.064^a^	.026 / .123	Complementary
	Perceived negative parenting experiences and compensatory behaviour mediated by Detached Self-Soother				
Emotionally depriving mother	.191^b^	.089	.102^a^	.052 / .173	Full
Conditional/narcissistic mother	.280^a^	.190^a^	.089^a^	.041 / .153	Complementary
Punitive mother	.216^a^	.136	.080^a^	.033 / .144	Full
Emotionally inhibited mother	.265^a^	.168^b^	.097^a^	.048 / .166	Complementary
Belittling mother	.242^a^	.134	.109^a^	.054 / .179	Full
Perfectionistic mother	.226^a^	.156^b^	.069^a^	.021 / .133	Complementary

^a^Significant at the 0.01 level

^b^Significant at the 0.05 level

## Discussion

To our knowledge, the current study is the first to explore the relationship between parenting, dysfunctional coping modes, and ED behaviours. Findings from this study highlight the role of the dysfunctional coping modes Perfectionistic Overcontroller, Self-Aggrandiser, Compliant Surrenderer, Detached Self-Soother and Detached Protector which partially explained the variance in the relationship between perceived negative parenting experiences and both restriction and compensatory behaviours in this sample. These findings build on previous research highlighting the potentially important relationship between coping processes and eating disordered behaviours [36, 45], and suggest that negative parenting behaviours that interfere with meeting the core emotional needs of childhood may not only be associated with the development of EMS but may also influence eating behaviours via the coping modes that the individual adopts.

The association between coping modes and both restricting and compensatory behaviour suggests that disordered eating is likely to fulfil a range of functions. Previous studies have suggested that eating disorder behaviours may function either as primary or secondary avoidance strategies, whereby individuals restrict, purge or overexercise in a compulsive or ritualised manner [7, 14, 21, 46] either as a means to reduce the chances of schema activation, or as a secondary strategy to block emotional distress that results from bingeing or other dietary transgressions [38, 39, 54]. Within the context of the Detached Protector/Self-Soother modes, restrictive and compensatory eating behaviours can function as a form of primary or secondary emotional *avoidance* whilst generating soothing feelings of numbness, or in some cases euphoria [23, 42]. In contrast, in Compliant Surrenderer mode, eating disordered behaviours may function as a form of passive compliance in deference to negative self-beliefs (associated with EMS) and the ED ‘voice’, thereby *surrendering* control to the disorder [32, 35].

Further, this study highlights the potential importance of the Perfectionistic Overcontroller mode, whereby via a focus on controllable behavior (i.e. restrictive eating rituals and perfectionism) the individual may *overcompensate* for underlying vulnerability and shame by replacing it with predictability, certainty, and a pseudo-sense of competence/self-worth [39, 53]. This supports previous research asserting the overcompensatory role of eating disordered behaviours (e.g. [6, 30]). Although the Self-Aggrandiser mode also functions as a form of overcompensation, this mode was endorsed at a relatively low level and only weakly correlated with eating behaviours, consistent with previous findings [45, 49].

Binge-eating was not found to correlate with perceived negative parenting, although this finding is likely to be a function of the lack of Bulimia Nervosa/binge eating in this sample.

These findings support previous theory suggesting the need to consider developmental factors and the function of ED behaviours in conjunction with maintenance factors, when conceptualising and treating EDs [36, 39, 45, 54]. The study also emphasises that treatment of EDs cannot rely solely on trait-based factors (i.e. EMS), and intervening with and understanding state factors (i.e. coping modes) should inform the conceptualisation of eating disorder symptoms. A major aspect of the Schema Mode Model is to challenge unhelpful messages that may be derived from early experiences and to build ‘Healthy Adult’ coping through a range of cognitive, behavioural, interpersonal and experiential techniques. Those with eating disorders are helped to recognise [previously unacknowledged] emotional needs, and to internalise a sense of self-compassion that enables them to begin to seek healthy relationships and interpersonal patterns, which thereby enables them to get these needs met. This is accomplished through a range of methods, including limited reparenting techniques, imagery rescripting, chair-work, and empathic confrontation [39].

Future studies in this area should extend the focus on schema modes to investigate the relationship between parenting, eating disorder behaviours and other types of modes (i.e. Parent and Child modes). Future studies should also explore the role of other negative experiences from childhood, including bullying, in the development of EMS, schema modes, and ED behaviours.

### Methodological considerations and limitations

A limitation of the current study is the fact that each of the behaviours (restriction, binging, purging and overexercising) were only measured with a single question, which may not adequately capture the complexity of these behaviours. In order to minimise participant fatigue, only the coping mode subscales of the SMI were included in this study. Moreover, the current study also re-included the Perfectionistic Overcontroller coping mode, despite it being excluded from the original SMI. Both of these issues may affect the reliability of the results. However, the fact that this emerged as an important mode in this study provides further support for future research to use the recently designed specialised SMI for EDs (SMI-ED), which has been validated with this population (Simpson S, Pietrabissa G, Rossi A, Frahn T, Manzoni M, Munro C, Nesci J, Castelnuovo G. Factorial Structure of the Schema Mode Inventory for Eating Disorders in a Clinical Sample, in preparation). Further, all data was gathered via self-report, which is subject to bias. While the cross-sectional nature of the study does not allow for causal attributions to be made, and perceptions of parenting styles are subjective, the current study nevertheless provides evidence that dysfunctional coping modes play a key role in the relationship between perceived negative parenting and ED psychopathology.

## Conclusions

Our findings suggest that Overcompensatory, Avoidant and Surrender coping mechanisms all appear to play a role in the maintenance of eating disorder symptoms, and that there are multiple complex relationships between these and EMS that warrant further investigation. This highlights the importance of early experiences and the potential role of early interventions for younger clients and their families as key to circumvent the development of EMS and dysfunctional coping modes that may lead to ED symptom development. The multitude of significant pathways between particular parenting styles, coping modes and restricting, bingeing and compensatory behaviour also highlight the complexity of ED psychopathology, including the way in which ED behaviours can fulfil multiple functions. This underlines the need to move beyond one-size-fits-all treatment approaches to more individualised approaches that can identify and work with the [historical] functional aspect of ED behaviours linked to coping modes, whilst highlighting their self-sabotaging role in the present. Schema Therapy facilitates the development of more effective and adaptive coping behaviours that directly focus on meeting emotional needs, whilst reducing reliance on ED coping behaviours. Future research that replicates the current findings, as well as research into the efficacy of schema-mode therapy for treating ED behaviours, is now warranted.
